# Differential response to biologics in a patient with severe asthma and ABPA: a role for dupilumab?

**DOI:** 10.1186/s13223-020-00454-w

**Published:** 2020-06-26

**Authors:** Carlo Mümmler, Bernd Kemmerich, Jürgen Behr, Nikolaus Kneidinger, Katrin Milger

**Affiliations:** 1 Department of Internal Medicine V, Ludwig-Maximilians-University of Munich (LMU), Marchioninistr.15, 81377 Munich, Germany; 2 Comprehensive Pneumology Center (CPC-M), Member of the German Center for Lung Research (DZL), Munich, Germany; 3 Pneumologie an der Münchner Freiheit, Munich, Germany

**Keywords:** ABPA, Aspergillosis, Asthma, Dupilumab, IL13, IL4

## Abstract

**Background:**

Allergic bronchopulmonary aspergillosis (ABPA) is a severe hypersensitivity reaction to aspergillus species colonizing the airways of patients with asthma or cystic fibrosis. Biologics including anti-IgE and anti-IL5 antibodies have strongly changed the treatment of severe asthmatics and have partly been reported to be effective in the treatment of ABPA. Recently, dupilumab, an anti-IL4-Rα antibody which inhibits signaling by the Th2-cytokines IL4 and IL13, has been approved for the treatment of severe asthma.

**Case presentation:**

Here, we report the case of a 49-year-old woman with severe asthma and ABPA, who was uncontrolled despite maximum inhalative therapy, anti-IL5-Rα antibody and continuous oral steroid therapy. Moreover, trials of itraconazole as well as omalizumab showed insufficient efficacy. Lung function revealed peripheral obstruction. FeNO and IgE were increased, eosinophils were suppressed under treatment while marked increases had been documented previously. Switching to dupilumab led to a complete resolution of pulmonary symptoms, resolution of exacerbations and complete withdrawal of oral steroids. A drastic improvement in lung function was noted, with an increase in FEV1 of almost 1 l. FeNO was normalized and IgE strongly reduced.

**Conclusion:**

Our case highlights that a patient may exhibit differential treatment responses to the currently available asthma biologics and suggests switching treatment if outcome is insufficient. A potential role for dupilumab in the treatment of ABPA warrants future studies.

## Background

Allergic bronchopulmonary aspergillosis (ABPA) is a hypersensitivity reaction to aspergillus species that predominantly affects cystic fibrosis and asthma patients [1]. Aspergillus colonization of the bronchial mucus leads to an activation of the innate immune system with a Th2-predominant T cell response [2–5]. Clinical features of ABPA comprise frequent asthma exacerbations, productive cough, airway obstruction, fever, and finally leads to end-stage lung disease. Diagnostic criteria are a predisposing condition, high total serum IgE levels, aspergillus-specific IgE or positive aspergillus skin test, aspergillus-specific IgG, peripheral blood eosinophilia and imaging findings consistent with ABPA [1, 6]. Clinical management involves oral corticosteroids and systemic therapy with azoles. In recent years, several studies and case reports showed efficacy for anti-IgE antibody and IL5/IL5-Rα antibodies in the treatment of ABPA [7–11]. Dupilumab is a novel IL4-Rα-antibody that has been approved for the treatment of atopic dermatitis, severe asthma and chronic rhinosinusitis with nasal polyps. Here, we describe a case of a pronounced treatment response to dupilumab in a patient with severe asthma and ABPA previously refractory to therapy.

## Case presentation

**Fig. 1:**
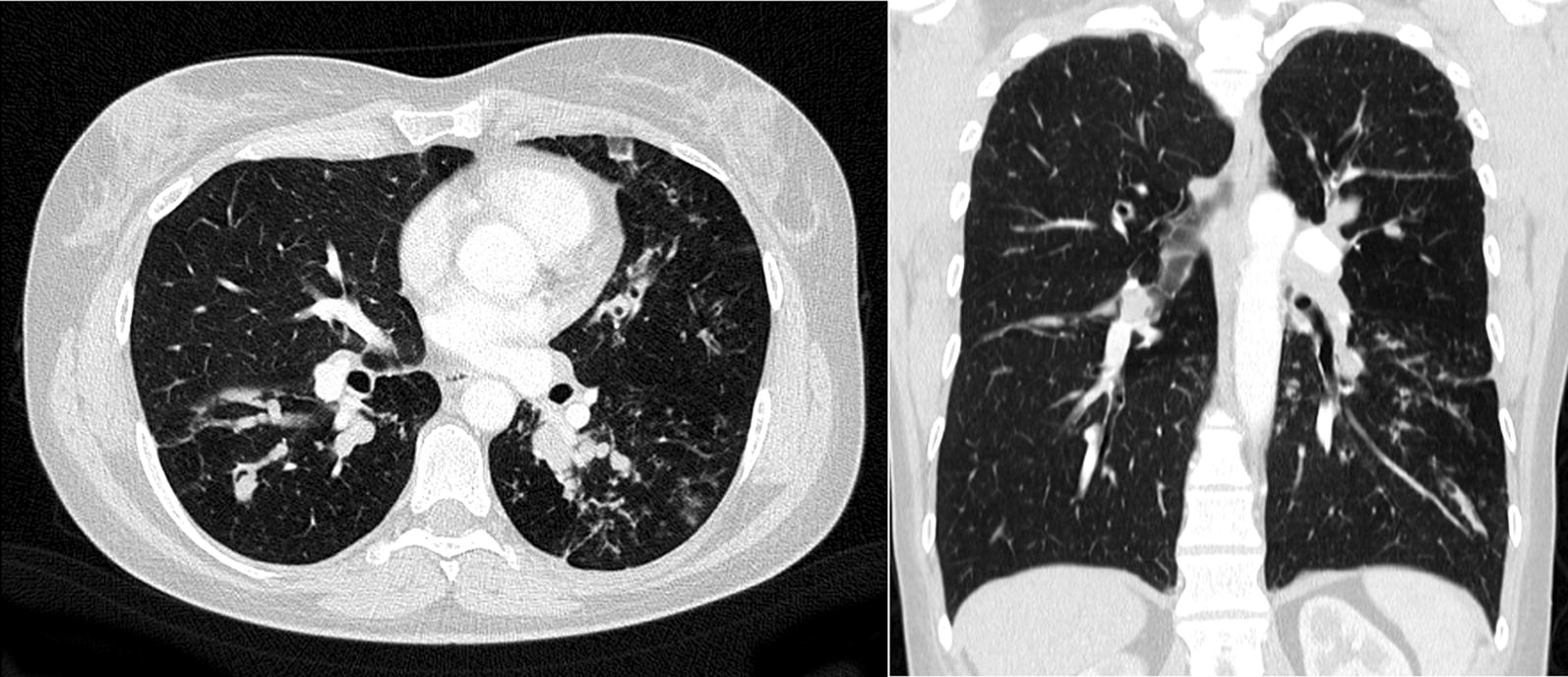
Chest CT demonstrating pulmonary infiltrates, bipulmonary bronchiectasis and bronchial wall thickening consistent with ABPA

A 49-year old female patient, never-smoker, presented to our outpatient clinic with a 20-year history of severe asthma. The patient reported poor asthma control with an asthma control test (ACT) score of 5/25 points. During the last 3 years her symptoms had deteriorated with increasing dyspnea, productive cough and nocturnal symptoms. She further experienced two asthma exacerbations during the last 12 months. At that time, her medication included high-dose ICS, LABA, oral prednisolone at 20 mg per day and anti-IL5-Rα treatment with benralizumab. She reported side effects from oral corticosteroid use including weight gain, osteopenia and steroid acne. Three years prior, she was diagnosed with ABPA based on bronchiectasis and pulmonary infiltrates (Fig. 1), eosinophilia of 950/µl (13%), total serum IgE of up to 7000 IU/ml, specific aspergillus IgE of 31.4 U/ml and specific aspergillus IgG of more than 200 mg/l. A trial of itraconazol had been performed but was stopped due to inefficacy. Pulmonary function testing during benralizumab showed peripheral obstruction with an FEV1 of 2.46 l (74% pred.), FEV1/FVC of 73%, small airway disease with MMEF of 1.76 l (50%) and increased RV of 157%. Fraction of exhaled nitric oxide (FeNO) was elevated at 56 ppb. Latest lab values during benralizumab revealed increased IgE of 6700 IU/ml and suppressed eosinophils of 0/µl. Benralizumab was discontinued after 6 months of treatment due to lack of improvement and no biologic was applied in the following 3 months. In this interval no change in symptoms was observed. As a potential therapeutic effect for omalizumab in the treatment of ABPA has been reported [9], a trial of omalizumab at 600 mg subcutaneously every 2 weeks was undertaken. However, again, no change in symptoms was seen after 5 months of treatment and omalizumab was stopped. Instead, 6 weeks later a trial of dupilumab was initiated with a loading dose of 600 mg and further 300 mg doses subcutaneously every 2 weeks.

**Fig. 2:**
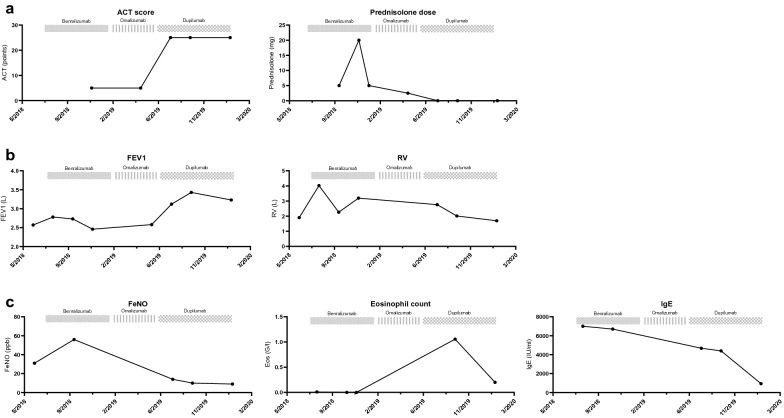
**a** Course of asthma control test (ACT) score and prednisolone dose over time. **b** Course of lung function parameters FEV1 and RV over time. **c** Course of FeNO, eosinophil count and IgE over time

At reevaluation 4 months after treatment initiation, the patient reported complete resolution of symptoms. ACT score had increased to 25/25 points. Oral steroid medication had been tapered completely and the patient experienced improvements of former steroid side effects (Fig. 2a). Lung function improved drastically with an FEV1-increase of almost 1000 ml to 3.43 l (99.6%), normalization of RV to 98% (Fig. 2b), and strong improvement in small airway obstruction (increase in MMEF from 1.76 to 2.87 l/s). FeNO decreased to a normal value of 10 ppb. Lung function improvements were sustained at 8 months follow-up (Fig. 2b). Laboratory analysis demonstrated a marked decrease in IgE to currently 940 IU/ml (reduction of 86%). Eosinophils at 4 months follow-up were increased to 1056/µl (16%), however normalized to 200/µl at 8 months follow-up (Fig. 2c). There were no changes in home or work environment during the reported period.

## Discussion

ABPA is a complication of bronchial asthma that renders disease management more difficult, often necessitating oral steroid treatment. High eosinophil counts are an important finding in the diagnosis of ABPA and initially eosinophils were markedly elevated in our patient. Thus, benralizumab had been started, which led to depletion of eosinophils, but did not improve symptoms, exacerbations or OCS dose. After discontinuation of benralizumab there was no change in symptoms underlining that the patient did not benefit from this treatment. Soeda et al. reported a case of asthma and ABPA with similar eosinophilia that responded well to benralizumab [11]. In contrast to our patient, that patient had less advanced disease without bronchiectasis and much lower total IgE of 537 IU/ml. Omalizumab decreased exacerbations in asthmatic patients with ABPA in a small prospective trial despite very high total IgE levels and a moderate omalizumab dose of 375 mg biweekly. However, improvement in lung function could not be demonstrated in that trial, and oral steroid tapering was not performed [9]. Our patient received a high dose of omalizumab (600 mg biweekly) but remained highly symptomatic and dependent on continuous OCS. In contrast, dupilumab led to a complete resolution of symptoms, discontinuation of OCS and normalization of lung function.

Eosinophils increased transiently after the initiation of dupilumab, however this was not associated with clinical symptoms and returned to a normal level during follow-up. In phase III trials of dupilumab in asthmatic patients, hypereosinophilia of more than 3000/µl was seen in 14% and 4.1% of dupilumab-treated patients, respectively and only led to discontinuation of dupilumab in few cases [12, 13]. It is hypothesized that dupilumab blocks the migration of eosinophils into (lung) tissues and therefore increases blood eosinophils transiently. The differential response to targeted biologic treatment suggests that in the present case the disease was mainly driven by IL4/IL13. Preclinical studies on APBA support a key role for IL4/IL13 signaling in ABPA. Dietschmann et al. showed that in a mouse model of ABPA, T-cells released huge amounts of IL4, IL5 and IL13 upon stimulation with *Aspergillus fumigatus* conidia. They further demonstrated that T cell-specific IL4/IL13-deficient-knockout mice exhibited reduced peripheral and lung eosinophilia, suggesting that IL4 and IL13 signaling leads to recruitment of eosinophils to the lung and blood from the bone marrow [3].

As comparative trials of different biologics in asthma and differential predictive biomarkers are currently lacking, guidelines recommend the trial of an antibody switch if the initial treatment did not show sufficient efficacy. This approach is supported by the presented case and previous treatment failures should not refrain clinicians from further trials of biologics that have different targets. It should be noted that the sequence in which the different biologics were applied here was mainly due to availability and licensing status of the drugs: dupilumab had only just become licensed for severe asthma when therapy was started in this patient.

## Conclusion

Altogether, this case report suggests that biological treatment should be switched to a drug with a different target, if treatment outcome under the previous biologic was insufficient. Further trials are needed to explore whether there is a general role for dupilumab in the treatment of ABPA.

## Patient perspective

*“After nearly 50* *years of breathlessness, therapy with dupilumab changed my life from one day to the next: to breathe without resistance is really a new quality of life! Perhaps there are two things in my case, which are special to this success. First, I am doing at minimum one hour of sport each day (bicycling, rowing) since 35* *years, even when this was hard to practice. And second: I lost, after starting with dupilumab and stopping cortisone, 17* *kg of weight within three months with a fasting cure. Altogether, this is giving me a reliable and hopeful perspective for my future.”*

## Data Availability

The data used during the current report are available from the corresponding author on reasonable request.

## Abbreviations

ABPA: Allergic broncho-pulmonary aspergillosis
Anti-IgE: Anti-immunoglobin E
Anti-Il4Rα: Anti-interleukin5-receptor-alpha
Anti-Il5Rα: Anti-interleukin5-receptor-alpha
FeNO: Fractional exhaled nitric oxide
FEV1: Forced expiratory volume in 1 s
OCS: Oral corticosteroid
RV: Residual volume
